# DDEC: Dual Dependency-Enhanced Contrastive Learning for Sparse Hypergraph Node Classification

**DOI:** 10.3390/e28070729

**Published:** 2026-06-25

**Authors:** Meilin Liu, Wenping Zheng, Shuxia Yuan

**Affiliations:** 1 School of Computer and Information Technology, Shanxi University, Taiyuan 030006, China; 202112407009@email.sxu.edu.cn (M.L.);; 2 Key Laboratory of Computational Intelligence and Chinese Information Processing of Ministry of Education, Shanxi University, Taiyuan 030006, China

**Keywords:** hypergraph neural networks, sparse hypergraph, node classification, dependency enhancement, contrastive learning

## Abstract

Hypergraph neural networks have shown strong potential for node classification due to their ability to capture high-order relationships and multi-granularity structural patterns. However, real-world hypergraphs are often sparse, which limits interaction modeling through node–hyperedge incidence and, in turn, weakens reliable attribute propagation and global dependency capture. To address this issue, we propose DDEC, a Dual Dependency-Enhanced Contrastive learning framework for sparse hypergraph node classification. To compensate for relational information lost under sparse structures, DDEC introduces an attribute view to complement the structural view. Since attribute information can be noisy and unreliable, we first design an entropy-guided feature recalibration mechanism to estimate node uncertainty and emphasize trustworthy attribute interactions. Building upon this, DDEC performs dual dependency enhancement from both structural and attribute perspectives. Specifically, we exploit the duality between a hypergraph and its line graph to perform line-graph transformation in both views, thereby constructing a shared dual relational space for interaction enhancement under sparse topologies. Within this dual space, we perform attention-based dependency enhancement in both views, so that the structural view captures explicit topological dependencies among hyperedges, while the attribute view uncovers latent semantic correlations beyond sparse incidence relations. The resulting representations from the two views are then adaptively fused, and collaborative contrastive learning is further performed at both the node and hyperedge levels to enforce multi-granularity semantic consistency. Experiments on eight public datasets demonstrate that DDEC consistently outperforms competitive baselines, validating its effectiveness and robustness.

## 1. Introduction

With the rapid proliferation of complex networked data, modeling high-order relationships among entities has become increasingly important in representation learning. Although conventional graphs have achieved notable success in a wide range of machine learning tasks, they are inherently limited to pairwise interactions and therefore often fall short in representing real-world systems involving multi-entity relations. Hypergraphs provide a natural and expressive alternative by allowing each hyperedge to connect an arbitrary number of nodes [1,2]. Owing to this advantage, hypergraph neural networks (HGNNs) have demonstrated strong effectiveness in various tasks, such as node classification, clustering, and recommendation [3,4,5].

Most existing HGNNs learn node representations through message passing over node–hyperedge incidence structures, where nodes aggregate high-order contextual information from their incident hyperedges. While this paradigm has achieved promising results, its effectiveness can deteriorate substantially when the underlying hypergraph is sparse. In many real-world scenarios, hypergraphs exhibit sparse connectivity [6], manifested as limited node–hyperedge interactions, weak hyperedge overlap, and fragmented structural organization. Such sparsity weakens the propagation of high-order information, reduces the reliability of local structural evidence, and hinders the modeling of long-range or implicit dependencies among hyperedges. As a consequence, node representations are often dominated by incomplete local observations, while unreliable attribute signals or structure-induced uncertainty may be propagated and amplified during message passing.

Despite the growing body of research on hypergraph learning, most existing methods still rely predominantly on local aggregation over the node–hyperedge bipartite structure [7]. This design becomes particularly vulnerable under sparse hypergraph settings for three reasons. First, sparse incidence patterns provide insufficient relational evidence for capturing dependencies beyond local neighborhoods, making it difficult to model latent interactions among hyperedges from structure alone. Second, most methods do not explicitly assess the reliability of attribute information. When raw features are weakly discriminative or incomplete, directly using them to construct auxiliary relations may introduce unreliable semantic neighbors, especially when structural support is weak. Third, semantic learning at different granularities is often handled in a decoupled manner, with limited coordination between node-level local consistency and hyperedge-level global semantics. These limitations call for a unified framework that can complement sparse structural evidence, strengthen trustworthy dependency modeling, and preserve semantic consistency across multiple granularities.

To this end, we propose DDEC, a Dual Dependency-Enhanced Contrastive learning framework for sparse hypergraph node classification. The central idea of DDEC is to improve representation learning by jointly exploiting structural and attribute information. When sparse incidence relations provide insufficient structural cues, the attribute view offers complementary semantic evidence for interaction modeling. Since attribute information may itself be noisy and unreliable, we first develop an entropy-guided feature recalibration mechanism to estimate node uncertainty and emphasize trustworthy attribute interactions. Building on this, we exploit the duality between a hypergraph and its line graph to perform line-graph transformation in both views, thereby constructing a shared dual relational space for interaction enhancement under sparse topologies. Within this dual space, attention-based dependency enhancement is performed in both views, so that the structural view captures explicit topological dependencies among hyperedges, while the attribute view uncovers latent semantic correlations beyond sparse incidence relations. The resulting representations from the two views are then adaptively fused. Furthermore, we design a collaborative contrastive learning strategy at both the node and hyperedge levels to enforce multi-granularity semantic consistency, thereby improving the robustness and discriminability of the learned representations.

Extensive experiments on eight public datasets show that DDEC consistently outperforms competitive baselines. Additional ablation and robustness analyses further validate the effectiveness and stability of the proposed framework.

The main contributions of this work are summarized as follows:

1. We propose DDEC, a unified framework for sparse hypergraph node classification that improves representation learning by jointly exploiting structural and attribute information.
2. We design an entropy-guided feature recalibration mechanism together with a dual-space dependency enhancement scheme to strengthen trustworthy interactions and improve dependency modeling under sparse hypergraph structures.
3. We develop a collaborative contrastive learning strategy at both node and hyperedge levels to enforce multi-granularity semantic consistency and enhance the robustness and discriminability of learned representations.
4. Extensive experiments on eight benchmark datasets, along with ablation and robustness studies, demonstrate the superiority, effectiveness, and stability of DDEC.

## 2. Related Work

Existing studies relevant to our work can be broadly grouped into two research lines: hypergraph neural networks and contrastive hypergraph learning. The former mainly focuses on high-order information propagation and dependency modeling over hypergraph structures, while the latter further introduces contrastive self-supervision to improve representation quality and semantic consistency.

### 2.1. Hypergraph Neural Networks

Hypergraph neural networks extend conventional graph learning beyond pairwise relations and provide a natural framework for modeling high-order interactions. Early studies mainly generalize graph convolution to node–hyperedge incidence structures. Representative models such as HGNN [7] establish the basic hypergraph propagation paradigm, while subsequent methods [8,9,10,11] improve it from different perspectives. UniGNN [12] further provides a unified formulation for graph and hypergraph neural networks. Although these methods improve hypergraph representation learning in various ways, they still largely rely on the original incidence structure and local propagation patterns.

To move beyond fixed propagation schemes, later studies explore more flexible structure modeling and adaptive hypergraph learning. For example, DHGNN [13] improves adaptability through dynamic hypergraph construction, DHKH [14] jointly models high-order and key structures, TDHNN [15] and HERALD [16] enhance representation quality through dynamic hyperedge modeling and Laplacian optimization, respectively, and MHSL [17] captures long-range dependencies via dynamic hypergraph construction. These methods enhance structural flexibility and better adapt hypergraph structures to downstream tasks. However, they still mainly refine node representations within the original or dynamically updated structure, with limited attention to explicit inter-hyperedge dependency modeling and semantic coordination across different granularities.

More recent studies seek to mitigate the challenges posed by sparse hypergraphs through auxiliary structural priors or explicit inter-hyperedge modeling. DPHGNN [18] incorporates low-order graph relations to alleviate feature degradation, and THTN [19] enhances cross-region interaction by introducing global nodes. Meanwhile, several methods explicitly model hyperedge relations beyond node-level aggregation. HeIHNN [20] constructs a hyperedge interaction graph, LHCN [21] performs hyperedge-level propagation through line-graph transformation, and HyperGT [22] and SHT [23] capture more complex hyperedge dependencies through global attention and learnable relation matrices, respectively. These studies highlight the importance of inter-hyperedge relation modeling when local node–hyperedge propagation becomes insufficient.

Nevertheless, existing methods still face several limitations in sparse settings. First, many approaches infer inter-hyperedge relations mainly through shared nodes or predefined structural rules, which restricts their ability to capture latent semantic dependencies beyond explicit structural overlap. Second, they generally do not provide a unified mechanism for jointly modeling topological and semantic dependencies when global structural evidence is fragmented. Third, most methods do not explicitly assess the reliability of propagated node features, making them vulnerable to noisy or uncertain signals when structural evidence is sparse. In contrast, DDEC combines uncertainty-aware feature recalibration with dual-space dependency enhancement, enabling it to suppress unreliable node signals while complementing incomplete inter-hyperedge dependencies in sparse hypergraphs.

### 2.2. Contrastive Hypergraph Learning

Contrastive learning has become an effective self-supervised paradigm for representation learning by enforcing consistency across different views and improving representation discriminability [24]. In hypergraph learning, it provides complementary supervision beyond labeled data and helps mitigate representation degradation caused by insufficient supervision and sparse structural connectivity.

Early contrastive hypergraph methods mainly focus on constructing multiple views for self-supervised optimization. For example, CCL [25] jointly exploits graph and hypergraph views, HyperGCL [26] combines perturbation-based and generative views, HSL [27] preserves consistency between optimized and original hypergraphs through intra-hyperedge contrast, and HCN [28] improves unsupervised hypergraph clustering via multi-view generation and structural alignment. These studies show that contrastive learning can provide useful supervisory signals beyond labels. However, many of them still emphasize node-level alignment, with limited explicit modeling of semantic consistency at the hyperedge level.

A subsequent line of work extends contrastive learning from node-level alignment to joint node–hyperedge modeling. TriCL [29], CHGNN [30], HyGCL-AdT [31], and HSCL [32] enhance high-order semantic modeling through node masking, hyperedge perturbation, multi-view augmentation, and hierarchical contrastive objectives. Although these methods provide richer self-supervised signals, they still rely substantially on augmentation-based view construction. Under sparse hypergraph settings, such perturbations may increase view diversity, but they may also further weaken high-order structural relations.

Another line of work further strengthens contrastive hypergraph learning by introducing structure-aware inductive biases. Specifically, H2SeqRec [33] models multi-scale temporal hypergraphs in hyperbolic space, OFSH [34] preserves core high-order semantics through key-node-guided adaptive augmentation, DHCN [35] improves sparse interaction modeling via line-graph transformation, and MCHNN [36] enhances representation learning under sparse association settings through multiple negative-sample hypergraphs. These methods demonstrate the benefit of integrating structural modeling with contrastive objectives, but they still address structural incompleteness and semantic consistency in a relatively fragmented manner.

Overall, existing contrastive hypergraph learning methods remain limited by insufficient multi-granularity contrastive modeling, perturbation-based view construction that may damage sparse structural relations, and inadequate exploitation of latent inter-hyperedge dependencies. In contrast, DDEC integrates uncertainty-aware feature recalibration, dual-space dependency enhancement, and collaborative contrastive learning at both the node and hyperedge levels within a unified framework. As a result, it improves robustness to noisy signals, complements fragmented inter-hyperedge dependencies, and promotes semantic consistency across local and global structural granularities.

## 3. Preliminaries

### 3.1. Hypergraph Definition and Problem Formulation

A hypergraph is a high-order generalization of an ordinary graph and can be represented as G=(V,E), where $V=\{v_{1},v_{2},\ldots ,v_{n}\}$ denotes the set of *n* nodes and $E=\{e_{1},e_{2},\ldots ,e_{m}\}$ denotes the set of *m* hyperedges. Different from a conventional graph in which each edge connects only two nodes, each hyperedge in a hypergraph can simultaneously connect an arbitrary number of nodes, thereby naturally modeling high-order relations. The topology of a hypergraph is commonly described by an incidence matrix $H\in R^{n\times m}$, where

(1) $$H_{ij}=\left\{\begin{array}{cc} 1, & \mathrm{if}\;v_{i}\in e_{j},\\ 0, & \mathrm{otherwise}. \end{array}\right.$$

Based on H, the degree of node $v_{i}$ and the cardinality of hyperedge $e_{j}$ are defined as $d\left(v_{i}\right)=\sum_{e_{j}\in E}H_{ij}$, $\delta \left(e_{j}\right)=\sum_{v_{i}\in V}H_{ij}$. Accordingly, the corresponding diagonal node-degree matrix and hyperedge-degree matrix are denoted by $D_{v}$ and $D_{e}$, respectively. Let $X\in R^{n\times d}$ denote the initial node feature matrix, where *d* is the feature dimension.

To describe the sparsity characteristics of a hypergraph from the incidence perspective, we further consider two descriptive statistics, namely the average node participation and the average hyperedge cardinality:

(2) $$\rho_{v}=\frac{1}{\left|V\right|}\sum_{v_{i}\in V}d\left(v_{i}\right),\;\rho_{e}=\frac{1}{\left|E\right|}\sum_{e_{j}\in E}\delta \left(e_{j}\right).$$

where $\rho_{v}$ measures the average number of hyperedges incident to each node, while $\rho_{e}$ measures the average number of nodes contained in each hyperedge. Although these two quantities are coupled through the total number of node–hyperedge incidences, they reflect sparsity from the node and hyperedge perspectives, respectively. Small values of $\rho_{v}$ and $\rho_{e}$ indicate limited node participation and restricted hyperedge scope, which together weaken high-order information propagation and make dependency modeling more challenging.

Given a partially labeled hypergraph G=(V,E) with node feature matrix X and a labeled node set $V_{L}\subset V$, the task of hypergraph node classification is to learn a prediction function f(G,X) that infers the labels of unlabeled nodes in $V\setminus V_{L}$. In sparse hypergraphs, the key challenge lies in learning discriminative node representations under insufficient structural interactions and unreliable dependency propagation.

### 3.2. Dual Line-Graph Transformation

**Figure 1:**
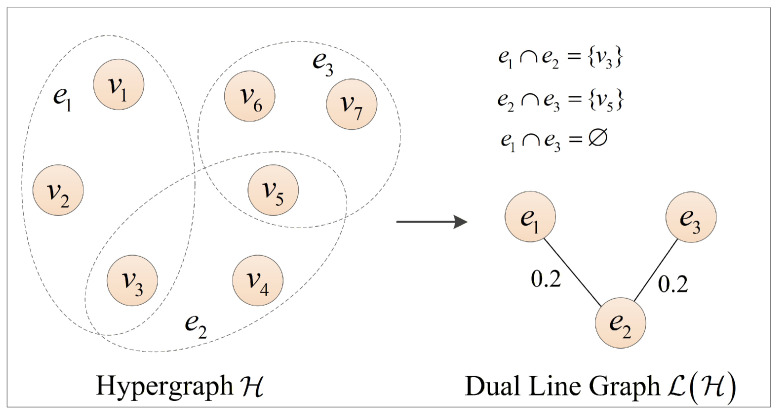
Illustration of a hypergraph and its dual line-graph transformation.

Given a hypergraph G=(V,E), its dual line graph is defined as $L\left(G\right)=\left(V_{L},E_{L}\right)$, where each node in $V_{L}$ corresponds to a hyperedge in E, i.e., $V_{L}=E$. Two dual nodes are connected if their corresponding hyperedges overlap. Formally, the edge set is defined as $E_{L}=\left\{\left(e_{i},e_{j}\right)|e_{i},e_{j}\in E,\;e_{i}\cap e_{j}\neq \emptyset ,\;i\neq j\right\}$. Thus, hyperedge overlap in the original hypergraph is converted into explicit adjacency in the dual line graph. As illustrated in Figure 1, this transformation maps each hyperedge to a node and represents overlap between hyperedges as graph connectivity.

To quantify the correlation strength between hyperedges, we adopt the Jaccard coefficient as the edge weight in the dual line graph:

(3) $$w_{ij}=\frac{|e_{i}\cap e_{j}|}{|e_{i}\cup e_{j}|},$$

where $w_{ij}$ measures the degree of overlap in node membership between hyperedges $e_{i}$ and $e_{j}$, and a larger value of $w_{ij}$ indicates a stronger topological association between them.

### 3.3. Hypergraph Convolution Propagation

Given a hypergraph G, a standard one-layer hypergraph convolution can be formulated as

(4) $$X^{\left(l+1\right)}=\sigma \left(D_{v}^{-\frac{1}{2}}HW_{e}D_{e}^{-1}H^{⊤}D_{v}^{-\frac{1}{2}}X^{\left(l\right)}\Theta^{\left(l\right)}\right),$$

where $\Theta^{\left(l\right)}$ is the learnable parameter matrix at layer *l*, $W_{e}$ is the hyperedge weight matrix, usually initialized as an identity matrix, σ(·) is a nonlinear activation function, and $X^{\left(l+1\right)}$ denotes the output node representations. According to Equation (4), the propagation first aggregates node features into the hyperedge space through $H^{⊤}$, performs information transformation at the hyperedge level, and then projects the updated hyperedge information back to the node space through H. In this way, a complete node–hyperedge–node message passing process is achieved, which provides the basic foundation for hypergraph representation learning.

## 4. Method

**Figure 2:**
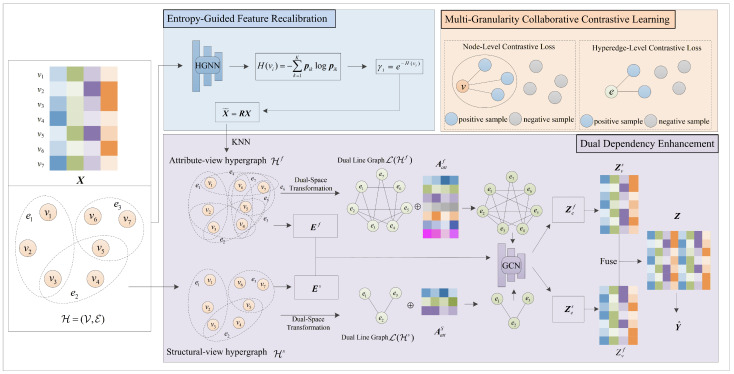
Framework of DDEC.

To address the challenges of sparse hypergraph node classification, we propose Dual Dependency-Enhanced Contrastive Learning (DDEC). As illustrated in Figure 2, DDEC consists of three key components: entropy-guided feature recalibration, dual dependency enhancement from the attribute and structural views, and multi-granularity collaborative contrastive learning. Specifically, DDEC first performs entropy-guided feature recalibration to suppress unreliable attribute signals under sparse structures. It then conducts dual dependency enhancement from both the attribute and structural views to complement missing relational information and model inter-hyperedge dependencies. Finally, a multi-granularity collaborative contrastive learning strategy is introduced to preserve semantic consistency and improve representation discriminability at both the node and hyperedge levels.

### 4.1. Entropy-Guided Feature Recalibration

In sparse hypergraphs, limited node–hyperedge interactions and weak hyperedge overlap make structural evidence insufficient for reliable representation learning. To compensate for missing relational information, we introduce an attribute view as a complementary source. However, raw node features may contain ambiguous, weakly discriminative, incomplete, or heterogeneous signals that cannot be fully resolved by the sparse structural context. Such unreliable signals may be amplified during message passing and impair subsequent attribute-based dependency modeling. This implicit noise is dataset-intrinsic rather than artificially injected and reflects the uncertainty in how much each node can reliably contribute to subsequent attribute interactions. To mitigate this effect, we perform entropy-guided feature recalibration before constructing the attribute view, suppressing unreliable signals and providing a more robust basis for dual dependency enhancement.

To estimate node-level feature reliability, we employ a lightweight auxiliary hypergraph predictor, which serves as an uncertainty estimator rather than the final classifier. The predictor consists of a single hypergraph convolution layer followed by a linear classifier and a softmax function. Given the node feature matrix X and the structural incidence matrix $H^{s}$, it first produces preliminary node representations through local hypergraph propagation and then generates the preliminary class distribution

$$P=\left[p_{ik}\right]\in R^{n\times K},$$

where *K* denotes the number of classes and $p_{ik}$ represents the probability that node $v_{i}$ belongs to class *k*. The auxiliary predictor is trained on labeled training nodes using the standard cross-entropy loss:

(5) $$L_{aux}=-\sum_{v_{i}\in V_{L}}\sum_{k=1}^{K}y_{ik}\log p_{ik},$$

where $V_{L}$ denotes the labeled training node set and $y_{ik}$ is the ground-truth label indicator. The parameters of the auxiliary predictor are independent of the main DDEC model and are not shared with the dual dependency enhancement module or the final classifier. Its output P is used only to compute node entropy for feature recalibration and does not directly determine the final node prediction.

It should be noted that DDEC does not require the auxiliary predictor to produce well-calibrated absolute class probabilities or highly accurate final predictions. Instead, the entropy derived from P is used as a relative reliability ranking signal. Nodes with flatter predictive distributions are regarded as relatively more uncertain, while nodes with more confident distributions are considered more reliable. Therefore, even though the auxiliary predictor may also be affected by sparse structures, its entropy output can still provide useful guidance for identifying relatively unreliable node signals before attribute-view construction. The entropy of node $v_{i}$ is defined as

(6) $$H\left(v_{i}\right)=-\sum_{k=1}^{K}p_{ik}\log p_{ik}.$$

A larger entropy indicates a flatter predictive distribution and thus a more uncertain class assignment, suggesting that the corresponding node features may contain stronger ambiguity or noise. In contrast, a smaller entropy implies higher predictive confidence and greater feature stability. Therefore, entropy serves as an effective indicator for assessing the relative reliability of node features under sparse structural conditions. To explicitly incorporate uncertainty into feature modeling, we convert the entropy of each node into a reliability weight through an exponential mapping:

(7) $$r_{i}=\exp\;\left(-H(v_{i})\right),$$

where $r_{i}$ denotes the reliability score of node $v_{i}$. This exponential mapping converts entropy into a positive reliability score and assigns exponentially smaller weights to highly uncertain nodes, thereby yielding a smooth and monotonic uncertainty-aware modulation. We then construct a diagonal reliability matrix $R=\mathrm{diag}(r_{1},r_{2},\ldots ,r_{n})$ and recalibrate the original node features as

(8) $$\tilde{X}=RX.$$

In this way, feature contributions are adaptively modulated according to node-specific uncertainty. Nodes with higher entropy are assigned smaller reliability weights to suppress potentially unreliable attribute signals, while nodes with lower entropy preserve more discriminative information. This node-wise recalibration reduces the adverse effect of noisy features before attribute-view construction and provides a more reliable basis for subsequent dual dependency enhancement and collaborative contrastive learning.

### 4.2. Dual Dependency Enhancement

To strengthen dependency modeling under sparse hypergraph structures, we perform dual dependency enhancement from both the structural and attribute views. This module consists of four stages: structural and attribute view construction, dual line-graph transformation with semantic affinity estimation, dependency enhancement with dual-space propagation, and confidence-aware multi-view fusion. Through these stages, DDEC enhances inter-hyperedge dependency modeling in the dual space and injects the enhanced relations back into node representations from two complementary views.

#### 4.2.1. Structural and Attribute View Construction

As the first step of dual dependency enhancement, we construct two complementary hypergraph views based on the recalibrated features to characterize observed topology and latent attribute affinity, respectively. The structural view directly inherits the original incidence structure with incidence matrix $H^{s}$ and preserves the observed node–hyperedge relations. In contrast, the attribute view is constructed from the recalibrated feature space to supplement sparse structural connectivity with feature-induced relational cues. It should be noted that the attribute view is not intended to replace the structural view, but to provide additional semantic evidence when the original node–hyperedge incidences are insufficient for reliable propagation.

Specifically, for each node $v_{i}$, we form an attribute-induced hyperedge by grouping $v_{i}$ together with its *k* nearest neighbors according to feature similarity in the recalibrated feature space, thereby obtaining the incidence matrix $H^{f}$. These attribute-induced hyperedges are not intended to replace the original hypergraph structure, but to provide complementary feature-level associations under sparse topology. In this way, the attribute view introduces additional relational cues beyond the original sparse structure and supports subsequent dependency enhancement.

Nevertheless, when raw node features are extremely weak or misleading, the constructed attribute view may still contain unreliable semantic neighbors, and entropy-guided recalibration alone may not fully remove such noise. To reduce this risk, DDEC does not rely solely on the attribute view. Instead, the structural and attribute views are later integrated through a confidence-aware gating mechanism, which adaptively adjusts their contributions. When the attribute view is less reliable, its influence can be down-weighted, allowing the model to rely more on the structural view.

For each view t∈{s,f}, the recalibrated node features are projected into the corresponding hyperedge space to obtain the initial hyperedge representations:

(9) $$E^{t}={\left(D_{e}^{t}\right)}^{-1}W_{e}^{t}{\left(H^{t}\right)}^{⊤}\tilde{X},$$

where $H^{t}$ is the incidence matrix of view *t*, $D_{e}^{t}$ is the corresponding hyperedge degree matrix, and $W_{e}^{t}$ is the hyperedge weight matrix. Equation (9) performs a weighted average aggregation of incident node features to initialize hyperedge representations in each view.

#### 4.2.2. Dual Line-Graph Transformation and Semantic Affinity Estimation

Based on the constructed structural and attribute views, we further enhance hyperedge dependency modeling in the dual space. For each view t∈{s,f}, we first transform the hypergraph into its dual line graph, where each hyperedge is mapped to a dual node and overlap between hyperedges is converted into explicit graph adjacency. In this way, local topological relations among hyperedges can be explicitly represented in the dual space.

However, explicit overlap-based adjacency alone is insufficient for sparse hypergraphs, since semantically related hyperedges may still be weakly connected or even disconnected in the original structure. To complement such missing relational cues, we further estimate latent semantic affinity among hyperedges through self-attention based on the initial hyperedge representations in Equation (9):

(10) $$A_{\mathrm{att}}^{t}=\mathrm{softmax}\left(\frac{Q\left(E^{t}\right)K{\left(E^{t}\right)}^{⊤}}{\sqrt{d_{k}}}\right),$$

where Q(·) and K(·) denote the query and key projection functions, respectively, and $d_{k}$ is the scaling factor.

Different from standard self-attention used for feature transformation, we retain only the normalized attention matrix as a semantic affinity estimator in the dual space. Larger values in $A_{\mathrm{att}}^{t}$ indicate stronger latent semantic correlations among hyperedges. As a result, each dual space contains two complementary types of relations: explicit topological adjacency induced by the line graph and implicit semantic affinity estimated from hyperedge representations.

#### 4.2.3. Dependency Enhancement and Dual-Space Propagation

After obtaining explicit dual topological adjacency and attention-based semantic affinity, we integrate them to enhance dependency modeling in sparse hypergraphs. Specifically, the enhanced dual adjacency matrix is constructed as

(11) $${\tilde{A}}^{t}=\alpha A_{\mathrm{top}}^{t}+\left(1-\alpha \right)\;{\mathrm{TopK}}_{s}\left(A_{\mathrm{att}}^{t}\right),$$

where $A_{\mathrm{top}}^{t}$ denotes the topological adjacency induced by the dual line graph, α is a balancing coefficient, and ${\mathrm{TopK}}_{s}\left(\cdot \right)$ retains the largest *s* entries in each row while setting the remaining entries to zero. This sparsification operation filters weak and potentially noisy semantic affinities, while preserving the most informative inter-hyperedge relations for sparse dependency enhancement.

The above fusion preserves explicit topological relations in the dual graph while supplementing them with attention-based semantic connections. Consequently, hyperedges that are weakly connected in topology but semantically related can still interact in the enhanced dual space, which facilitates dependency modeling beyond local structural overlap.

Given the enhanced dual adjacency matrix, we update hyperedge representations in the dual space via graph convolution:

(12) $$Z_{e}^{t}=\sigma \left({\left({\tilde{D}}^{t}\right)}^{-\frac{1}{2}}{\tilde{A}}^{t}{\left({\tilde{D}}^{t}\right)}^{-\frac{1}{2}}E^{t}\Theta_{e}^{t}\right),$$

where ${\tilde{D}}^{t}$ is the degree matrix of ${\tilde{A}}^{t}$, $\Theta_{e}^{t}$ is a learnable parameter matrix, and σ(·) is the nonlinear activation function. Since the dual line graph treats hyperedges as nodes, graph convolution in the dual space enables explicit propagation of inter-hyperedge dependencies that cannot be directly captured in the original incidence space.

The enhanced hyperedge representations are then projected back to the node space to obtain view-specific node representations:

(13) $$Z_{v}^{t}=\sigma \left({\left(D_{v}^{t}\right)}^{-1}H^{t}Z_{e}^{t}\Theta_{v}^{t}\right),$$

where $D_{v}^{t}$ is the node degree matrix of view *t* and $\Theta_{v}^{t}$ is the projection parameter matrix. Through this projection, the enhanced dependencies learned in the dual space are injected back into node representations for each view.

#### 4.2.4. Confidence-Aware Multi-View Fusion

After dual dependency enhancement in the structural and attribute views, we obtain view-specific node representations $Z_{v}^{s}$ and $Z_{v}^{f}$. Since these two views provide complementary information, we further design a confidence-aware gated fusion mechanism to adaptively integrate them. Different from the entropy-based reliability used for input recalibration, the confidence defined here is view-specific and is used to estimate the predictive quality of each view for adaptive fusion.

First, the node representations from each view are mapped to the label space to obtain the corresponding predictive probability matrices:

(14) $$P^{t}=\mathrm{softmax}\left(Z_{v}^{t}W_{c}+b\right),\;t\in \left\{s,f\right\},$$

where $W_{c}$ is a shared learnable mapping matrix, b is a bias term, and the number of classes is *K*. The shared mapping projects both views into the same label space, making their prediction scores comparable for confidence estimation.

To assess the reliability of each view at the prediction level, we define a confidence vector by jointly considering the maximum class probability and the margin between the largest and second-largest probabilities:

(15) $$c^{t}=\max\left(P^{t}\right)+\left(\max\left(P^{t}\right)-2\mathrm{ndmax}\left(P^{t}\right)\right),$$

where $\max(P^{t})$ and $2\mathrm{ndmax}(P^{t})$ denote the row-wise largest and second-largest values of $P^{t}$, respectively. This definition jointly reflects absolute prediction confidence and relative class separation, thereby providing a simple yet effective estimate of view-specific reliability.

The confidence scores from the two views are normalized to obtain the corresponding gating weights:

(16) $$G^{t}=\frac{c^{t}}{c^{s}+c^{f}},\;t\in \left\{s,f\right\}.$$

The resulting gating weights are node-wise scalar coefficients, which are broadcast along the feature dimension to modulate the contribution of each view. Based on these weights, the final fused node representation is given by

(17) $$Z=G^{s}⊙Z_{v}^{s}+G^{f}⊙Z_{v}^{f},$$

where ⊙ denotes the Hadamard product.

Finally, the fused node representation is fed into a two-layer multilayer perceptron followed by a softmax classifier to produce the final prediction:

(18) $$\hat{Y}=\mathrm{softmax}\left(\mathrm{MLP}\left(Z\right)\right).$$

### 4.3. Multi-Granularity Collaborative Contrastive Learning

Although the preceding modules provide more reliable node representations and enhanced dependency modeling, supervised classification alone is still insufficient to constrain semantic consistency across different granularities in sparse hypergraphs. In particular, sparse topology may weaken local contextual coherence at the node level and impair the stability of global dependency semantics at the hyperedge level. To address this issue, we introduce a multi-granularity collaborative contrastive learning strategy that imposes complementary self-supervised constraints on both nodes and hyperedges. In this way, local semantic consistency and global dependency consistency are jointly preserved to further improve the robustness and discriminability of the learned representations.

#### 4.3.1. Node-Level Contrastive Loss

To preserve local semantic consistency under sparse structural conditions, we first introduce a node-level contrastive objective on the fused node representations. Let Z denote the fused node representation matrix. For a node $v_{i}$, we define its positive sample set $P_{i}$ as the set of nodes that share at least one hyperedge with $v_{i}$, and its negative sample set $N_{i}$ as the set of nodes that have no direct hyperedge association with $v_{i}$. Based on the InfoNCE formulation, the node-level contrastive loss is defined as

(19) $$L_{\mathrm{node}}=-\sum_{v_{i}\in V}\log\frac{\sum_{v_{j}\in P_{i}}\exp\;\left(\mathrm{sim}(z_{i},z_{j})/\tau \right)}{\sum_{v_{j}\in P_{i}}\exp\;\left(\mathrm{sim}(z_{i},z_{j})/\tau \right)+\sum_{v_{k}\in N_{i}}\exp\;\left(\mathrm{sim}(z_{i},z_{k})/\tau \right)},$$

where sim(·,·) denotes cosine similarity and τ is the temperature coefficient. This objective encourages nodes sharing hyperedge context to remain close in the embedding space, thereby strengthening local semantic coherence and improving the stability of node representations under sparse topology.

#### 4.3.2. Hyperedge-Level Contrastive Loss

Beyond node-level local consistency, sparse hypergraph learning also requires preserving dependency-aware semantic relations among hyperedges. After dual dependency enhancement, hyperedges that are strongly connected in the enhanced dual space should remain close in the representation space. To this end, we further introduce a hyperedge-level contrastive objective to stabilize global dependency semantics.

Let $Z_{e}^{t}$ denote the hyperedge representation matrix in the dual space for view t∈{s,f}. For each view, two hyperedges are treated as a positive pair if they are connected in the enhanced dual adjacency matrix ${\tilde{A}}^{t}$, and as a negative pair otherwise. Accordingly, the hyperedge-level contrastive loss is defined as

(20) $$L_{\mathrm{edge}}=-\sum_{e_{i}\in E}\log\frac{\sum_{e_{j}\in P_{i}^{e}}\exp\;\left(\mathrm{sim}(e_{i},e_{j})/\tau \right)}{\sum_{e_{j}\in P_{i}^{e}}\exp\;\left(\mathrm{sim}(e_{i},e_{j})/\tau \right)+\sum_{e_{k}\in N_{i}^{e}}\exp\;\left(\mathrm{sim}(e_{i},e_{k})/\tau \right)},$$

where sim(·,·) denotes cosine similarity, and $P_{i}^{e}$ and $N_{i}^{e}$ denote the positive and negative hyperedge sets associated with hyperedge $e_{i}$, respectively. This objective encourages hyperedges with strong topological or semantic dependencies in the enhanced dual space to stay close in the embedding space, thereby preserving global dependency consistency and reducing semantic drift caused by sparse structural fragmentation.

#### 4.3.3. Joint Optimization Objective

We jointly optimize the supervised classification objective together with the two contrastive objectives. The overall loss function is defined as

(21) $$L=L_{\mathrm{CE}}+\lambda_{1}L_{\mathrm{node}}+\lambda_{2}L_{\mathrm{edge}},$$

where $L_{\mathrm{CE}}$ is the cross-entropy classification loss, and $\lambda_{1}$ and $\lambda_{2}$ are hyperparameters that control the strengths of the node-level and hyperedge-level contrastive objectives, respectively.

Through joint optimization, the node-level contrastive objective preserves local semantic consistency, while the hyperedge-level contrastive objective stabilizes global dependency semantics. Together, they complement supervised classification and further improve the robustness and discriminability of representations learned on sparse hypergraphs.

### 4.4. Algorithmic Procedure

The overall procedure of DDEC is summarized in Algorithm 1. Given the original hypergraph and node feature matrix, DDEC first performs entropy-guided feature recalibration to obtain more reliable node features. Based on the recalibrated features, it then constructs structural and attribute views, enhances hyperedge dependencies in the dual space for each view, and adaptively fuses the resulting node representations through confidence-aware gating. Finally, node-level and hyperedge-level contrastive objectives are jointly optimized together with the classification loss to learn robust and discriminative representations for sparse hypergraph node classification.

### 4.5. Time Complexity Analysis

To analyze the computational efficiency of DDEC, we summarize the time complexity of its main components. Let *n* denote the number of nodes, *d* the hidden dimension, *c* the number of classes, and $m^{s}$ and $m^{f}$ the numbers of hyperedges in the structural and attribute views, respectively. Let $|I^{s}|$ and $|I^{f}|$ denote the numbers of node–hyperedge incidences in the two views. We use *q* to denote the number of candidate hyperedge neighbors used for semantic affinity estimation and *s* to denote the number of retained neighbors after sparsification.

The entropy-guided feature recalibration module obtains preliminary predictions through a lightweight auxiliary hypergraph predictor, with complexity $O(|I^{s}|d+ndc)$. Entropy estimation and feature recalibration further require O(nc+nd). The structural view is directly inherited from the original incidence matrix without extra construction cost. The attribute view is constructed once from recalibrated node features using a *k*-nearest-neighbor strategy, which costs $O(n^{2}d)$ in a straightforward implementation. Since this step is performed only once before training, it is treated as preprocessing and excluded from the per-epoch complexity.
**Algorithm 1:** The overall procedure of DDEC**Input**: Hypergraph G, node feature matrix X, balancing coefficient α, neighborhood size *k*, sparsification parameter *s*, temperature τ, loss weights $\lambda_{1}$ and $\lambda_{2}$**Output**: Predicted node labels**1** Obtain the preliminary predictive distribution P using an auxiliary hypergraph predictor;**2** Compute node entropy $H(v_{i})$ according to Equation (6);**3** Construct the reliability matrix R and recalibrate node features $\tilde{X}$ via Equation (8);**4** Inherit the structural view $H^{s}$ from the original incidence matrix and construct the attribute view $H^{f}$ based on $\tilde{X}$; Project $\tilde{X}$ into the hyperedge spaces of the two views to obtain initial hyperedge representations via Equation (9);**5** For each view, transform the hypergraph into its dual line graph and construct the dual topological adjacency matrix;**6** Estimate the hyperedge semantic affinity matrix according to Equation (10);**7** Fuse explicit topology and semantic affinity to obtain the enhanced dual adjacency matrix via Equation (11);**8** Update hyperedge representations in the dual space using Equation (12);**9** Project the enhanced hyperedge representations back to the node space via Equation (13);**10** Compute the confidence scores and gating weights according to Equations (15) and (16), and fuse the two-view node representations via Equation (17);**11** Compute the node-level contrastive loss $L_{\mathrm{node}}$ and the hyperedge-level contrastive loss $L_{\mathrm{edge}}$ according to Equations (19) and (20);**12** Optimize the overall objective in Equation (21) and update model parameters;**13** Output the final node predictions according to Equation (18);

For each view t∈{s,f}, projecting node representations into the hyperedge space and projecting enhanced hyperedge representations back to the node space cost $O(|I^{t}|d)$. In dual dependency enhancement, semantic affinity is estimated only within a candidate neighbor set of size *q*, rather than by dense all-pair computation, leading to complexity $O(m^{t}qd)$. After top-*s* sparsification, dual-space graph convolution is performed on the sparse enhanced adjacency matrix with complexity $O(m^{t}sd)$. The confidence-aware two-view fusion costs O(nd).

For optimization, the supervised classification loss costs O(nc). The node-level contrastive loss is computed over sparse incidence-induced positive pairs, with complexity $O((|I^{s}\left|+\right|I^{f}\left|)d\right)$. The hyperedge-level contrastive loss is computed over retained dual-space neighbors, with complexity $O(\left(m^{s}s+m^{f}s\right)d)$.

Therefore, excluding the one-time attribute-view preprocessing cost, the per-epoch time complexity of DDEC can be summarized as

(22) $$O\left(ndc+\sum_{t\in \{s,f\}}\left(|I^{t}|d+m^{t}qd+m^{t}sd\right)\right).$$

Since *q* and *s* are much smaller than the number of hyperedges in sparse hypergraphs, DDEC avoids dense hyperedge-level dependency computation and mainly operates on sparse incidence matrices and sparse dual-space adjacency matrices.

## 5. Experimental Results and Analysis

### 5.1. Experimental Settings

To comprehensively evaluate the effectiveness of DDEC for sparse hypergraph node classification, we conduct experiments on eight public datasets drawn from diverse real-world scenarios, including academic networks, biological data, social networks, and e-commerce/multimedia interaction networks. These datasets exhibit substantial diversity in terms of structural sparsity, feature dimensionality, and class cardinality, thereby providing a comprehensive test bed for assessing the generalizability and robustness of the proposed framework across different sparse hypergraph scenarios. The detailed statistics of all datasets are reported in Table 1.

To verify the effectiveness of the proposed framework, we compare DDEC against 10 representative hypergraph learning baselines spanning three categories: classical hypergraph neural networks, dynamic structure modeling methods, and contrastive-learning-based hypergraph representation learning methods. Specifically, the classical baselines include HGNN, HNHN, and AllSet; the dynamic structure modeling baselines include DHGNN and TDHNN, and the recent hypergraph method DPHGNN, which we include as an additional baseline using hyperedges reconstructed from our datasets to ensure fair comparison; the contrastive baselines include HyperGCL, HSL, CHGNN, HyGCL-AdT, and we note that HyperGT is not included because its original experimental setting focuses on featureless hypergraphs, which differs from our attributed sparse hypergraph node classification scenario and would require nontrivial adaptation. This broad selection enables a comprehensive evaluation of DDEC against different families of competitive methods.

We adopt classification accuracy (ACC) as the evaluation metric. For each dataset, the training, validation, and test sets are split according to a ratio of 1:1:8. To ensure a fair comparison, all baseline methods are trained and evaluated under the same data split.

Unless otherwise specified, the key hyperparameters of DDEC are set as follows. The number of nearest neighbors used to construct the attribute-view hypergraph is fixed at k=10. In the dual dependency enhancement module, the fusion coefficient between explicit topological adjacency and attention-derived semantic affinity is set to α=0.6, and the sparsification threshold for semantic affinity is set to s=5. In the joint optimization objective, the weights of the node-level and hyperedge-level contrastive losses are set to $\lambda_{1}=0.3$ and $\lambda_{2}=0.4$, respectively.

For the entropy-guided feature recalibration module, the auxiliary predictor is a lightweight hypergraph convolutional network consisting of a single hypergraph convolution layer followed by a linear classifier and softmax function to generate preliminary class distributions P. It is trained on the same training split using standard cross-entropy loss. The parameters of the auxiliary predictor are independent of the main DDEC model and are not shared with other modules. Its output is only used to compute node-level predictive entropy to guide feature recalibration; it does not directly participate in final prediction. Therefore, the auxiliary predictor serves as a fixed uncertainty estimator rather than an additional prediction branch in the main model. Its contribution to final performance is confirmed in the ablation study in Section 5.3.

All experiments are implemented in PyTorch 2.3.1 and conducted on a single NVIDIA A100 GPU. To reduce the influence of randomness, each experiment is repeated five times with different random seeds, and the reported results are averaged over these runs. Baselines follow the parameter settings recommended in their original papers and are further tuned on validation sets to ensure fair comparison.

### 5.2. Overall Performance Comparison

Table 2 reports the node classification accuracy of DDEC and all baseline methods on eight public datasets. Overall, DDEC achieves superior or competitive performance across all datasets, obtaining the best results on five datasets: Cora-C, Citeseer, Cora-A, Zoo, and Amazon. These results demonstrate the effectiveness of DDEC for sparse hypergraph node classification across diverse real-world scenarios.

More specifically, DDEC outperforms the strongest competing baseline by 1.31, 2.42, 0.92, 1.48, and 0.38 percentage points on Cora-C, Citeseer, Cora-A, Zoo, and Amazon, respectively. The gains on Citeseer, Zoo, and Cora-C are particularly notable. According to the statistics in Table 1, Cora-C, Citeseer, and Cora-A have relatively high intra-hyperedge label homophily ratios, with h=0.8625, 0.8291, and 0.8763, respectively. This indicates that nodes within the same hyperedge tend to share relatively coherent class semantics, making node-level consistency modeling more reliable. In addition, Zoo has a much larger average number of neighboring hyperedges in the dual space, with ζ=5.58, suggesting that rich inter-hyperedge dependencies remain recoverable even though its intra-hyperedge homophily is not very high. These observations indicate that DDEC can benefit from either reliable local semantic consistency or recoverable global dependency structures, especially when sparse high-order interactions are only weakly observed from the original topology.

On Mushroom, DDEC performs slightly worse than the best baseline. Although Mushroom has a high intra-hyperedge label homophily ratio (h=0.9554), its average number of neighboring hyperedges in the dual space is extremely small (ζ=0.09), indicating that there are very limited inter-hyperedge associations to recover. Under such conditions, the effectiveness of dual dependency enhancement is constrained, and the relational supervision provided by the contrastive objectives is also weakened. This explains why DDEC only achieves limited improvement on this dataset despite its strong local homophily.

DDEC also does not achieve the best result on Pokec and Actor. From Table 1, both datasets have relatively low dual-space connectivity and weaker intra-hyperedge semantic consistency. Specifically, Pokec has ζ=0.66 and h=0.7225, while Actor has ζ=0.31 and h=0.6755. These statistics indicate that the recoverable inter-hyperedge dependencies are limited and that nodes within the same hyperedge may not share highly consistent class semantics. For example, in Pokec, a hyperedge corresponds to a friendship group whose members may differ in gender, interests, and other attributes. In Actor, a hyperedge corresponds to a movie project and may include actors, directors, and screenwriters with different roles. In such cases, enforcing node-level local consistency may introduce ambiguous supervision and blur the original class boundaries, thereby reducing classification performance.

Overall, the results suggest that DDEC is most effective when useful inter-hyperedge dependencies remain recoverable in the dual space, and relatively high intra-hyperedge homophily can further enhance the benefit of node-level consistency modeling. When dual-space connectivity is extremely weak, as in Mushroom, the benefit of dual dependency enhancement becomes limited even if local homophily is high. When both dual-space connectivity and intra-hyperedge semantic consistency are relatively weak, as in Pokec and Actor, the advantage of DDEC is also reduced. These observations provide more concrete guidance for applying DDEC: it is particularly suitable for sparse hypergraphs with recoverable global dependency structures, while reliable local contextual consistency can further improve its stability.

### 5.3. Ablation Study

**Figure 3:**
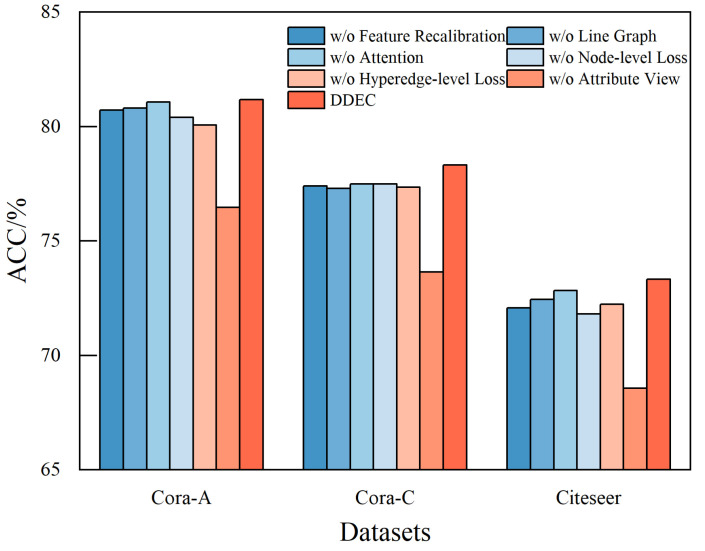
Ablation study of the core components in DDEC for sparse hypergraph node classification.

To investigate the contribution of each component in DDEC, we conduct ablation studies on Cora-A, Cora-C, and Citeseer. Six model variants are constructed by removing one component at a time, including feature recalibration, dual line-graph transformation, attention-based semantic enhancement, node-level contrastive learning, hyperedge-level contrastive learning, and the attribute view. The variant definitions are listed in Table 3, and the corresponding results are shown in Figure 3.

Overall, the full DDEC model achieves the best performance on all three datasets, which demonstrates that the proposed components contribute to sparse hypergraph node classification in a highly complementary manner. In contrast, removing any single component leads to a noticeable performance drop, indicating that uncertainty-aware feature recalibration, dual dependency enhancement, and multi-granularity collaborative contrastive learning all play important roles in the final performance gains.

Specifically, removing the feature recalibration module decreases accuracy by 0.46, 0.92, and 1.24 percentage points on Cora-A, Cora-C, and Citeseer, respectively. This module relies on the lightweight auxiliary hypergraph predictor, which is trained independently and used to generate preliminary class distributions for computing node-level entropy. The entropy serves as a relative reliability signal, suppressing unreliable node features before constructing the attribute view. The observed performance drop confirms that this entropy-guided feature recalibration effectively mitigates the impact of noisy or ambiguous node signals, providing more stable inputs for subsequent dependency modeling.

For the dual dependency enhancement module, removing the line graph, attention mechanism, or attribute view consistently degrades performance, indicating that jointly modeling explicit topological adjacency and latent semantic affinity is critical for recovering incomplete inter-hyperedge dependencies in sparse hypergraphs. Among these variants, removing the attribute view causes the largest drop, with accuracy decreasing by 4.70, 4.66, and 4.75 percentage points on the three datasets, respectively. This suggests that the attribute view provides crucial supplementary relational cues beyond the sparse structural view, especially when high-order interactions are only weakly observed from topology alone.

For the collaborative contrastive learning module, removing the node-level contrastive loss results in accuracy drops of 0.78, 0.83, and 1.51 percentage points, while removing the hyperedge-level contrastive loss leads to decreases of 1.11, 0.97, and 1.09 percentage points on the three datasets, respectively. These results indicate that the node-level contrastive objective helps preserve local semantic consistency among structurally related nodes, whereas the hyperedge-level contrastive objective stabilizes global dependency semantics in the dual space. Together, the two objectives provide complementary supervision from local node relations and global hyperedge dependencies, thereby improving the discriminability and stability of the learned representations.

In summary, the ablation results show that the performance gains of DDEC do not arise from any single design choice, but from the joint effect of uncertainty-aware feature recalibration, dual dependency enhancement, and multi-granularity collaborative contrastive learning. In particular, the attribute view and dual-space dependency enhancement help recover incomplete inter-hyperedge dependencies under sparse topology, while node-level and hyperedge-level contrastive learning further preserve local semantic consistency and stabilize global dependency semantics.

### 5.4. Parameter Sensitivity Analysis

To further investigate the influence of key hyperparameters, we conduct a sensitivity analysis on the Citeseer dataset. Specifically, we examine the fusion coefficient α and the sparsification threshold *s* in the dual dependency enhancement module, as well as the loss weights $\lambda_{1}$ and $\lambda_{2}$ in the joint optimization objective.

**Figure 4:**
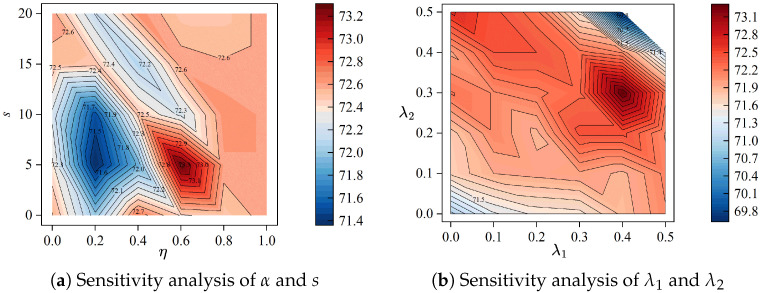
Sensitivity analysis of key parameters.

Figure 4a illustrates the classification accuracy under different combinations of α and *s*, where s∈[0,20]. The results show that model performance is sensitive to both hyperparameters. When α is too large, the model relies excessively on explicit topological adjacency and fails to fully exploit latent semantic affinity among hyperedges. In contrast, when α is too small, the structural constraints inherited from the original hypergraph become insufficient. Similarly, when *s* is too small, only a limited number of meaningful semantic connections are retained. When *s* is too large, weak and noisy relations are more likely to be introduced. The best performance is achieved at α=0.6 and s=5, indicating that explicit topological adjacency and attention-derived semantic affinity should be properly balanced, and that retaining only a small number of high-confidence semantic links is most beneficial for dual-space dependency enhancement.

Figure 4b presents the heat map of classification accuracy under different values of $\lambda_{1}$ and $\lambda_{2}$. When both weights are too small, the node-level and hyperedge-level contrastive losses are too weak to provide effective auxiliary supervision for representation learning. In contrast, when both are too large, the contrastive objectives dominate the optimization process and weaken the contribution of the supervised classification signal, leading to performance degradation. The best result is obtained when $\lambda_{1}=0.3$ and $\lambda_{2}=0.4$. This suggests that the node-level and hyperedge-level contrastive objectives should be balanced appropriately with the classification objective in order to fully exploit their complementary roles in preserving local semantic consistency and stabilizing global dependency semantics.

### 5.5. Robustness Analysis

**Figure 5:**
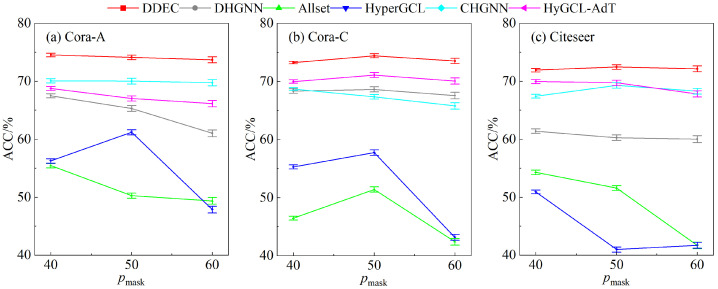
Robustness comparison of DDEC and baseline methods under structural perturbation.

**Figure 6:**
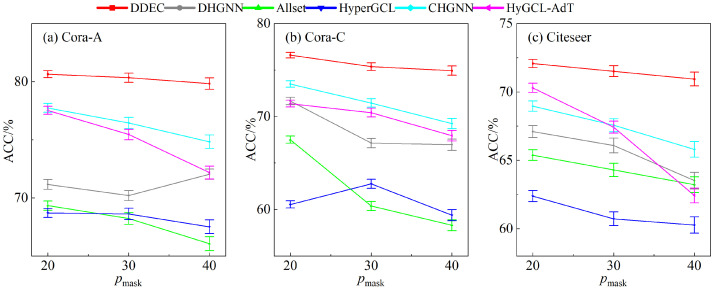
Robustness comparison of DDEC and baseline methods under feature perturbation.

To evaluate the robustness of DDEC under sparse perturbations, we conduct robustness experiments on Cora-A, Cora-C, and Citeseer from two perspectives: structural perturbation and feature perturbation. DDEC is compared with representative methods, including HGNN and CHGNN, and classification accuracy is used as the evaluation metric. In the structural robustness experiments, 40%, 50%, and 60% of node–hyperedge incidence relations are randomly removed, followed by the deletion of 70% of hyperedges to simulate different levels of structural damage. In the feature robustness experiments, partial node features are randomly masked and set to zero, with masking ratios $p_{\mathrm{mask}}\in \left\{20\%,30\%,40\%\right\}$. The results are shown in Figure 5 and Figure 6, respectively.

As shown in Figure 5, although the performance of all methods fluctuates as structural perturbation becomes stronger, DDEC consistently remains superior on Cora-A, Cora-C, and Citeseer. This demonstrates that DDEC has strong robustness under structural corruption. Such robustness can be attributed to three factors. First, uncertainty-aware feature recalibration suppresses unreliable signal propagation. Second, dual dependency enhancement helps recover incomplete inter-hyperedge dependencies under sparse topology. Third, collaborative contrastive learning further preserves node-level local semantic consistency and stabilizes hyperedge-level global dependency semantics. As a result, DDEC remains stable even under severely corrupted structural conditions.

Figure 6 further shows that, as feature perturbation increases, the classification accuracy of all methods generally declines. Nevertheless, DDEC still outperforms the competing baselines on all three datasets, indicating that it is also robust to feature corruption. This result suggests that the proposed feature recalibration effectively alleviates the interference caused by low-confidence features, while the attribute view and confidence-aware multi-view fusion help compensate for missing or corrupted node attributes by exploiting complementary feature-induced relational cues. Consequently, DDEC maintains strong classification capability even when the input features are partially damaged.

### 5.6. Training Time Comparison

To further evaluate the practical efficiency of DDEC, we report the wall-clock training time of DDEC and two representative baselines, HyGCL-AdT and DPHGNN, on five datasets. All methods are evaluated under the same experimental environment, and the results are shown in Table 4.

As shown in Table 4, DDEC generally requires more training time than simpler baselines on large-scale datasets. This is mainly because DDEC introduces dual line-graph transformation, hyperedge-level semantic affinity estimation, and node-level and hyperedge-level contrastive learning. Nevertheless, the additional cost is expected and remains manageable on modern GPUs. Moreover, DDEC can be successfully trained on all datasets, indicating its practical feasibility for sparse hypergraph node classification.

## 6. Conclusions and Future Work

In this paper, we propose DDEC, a Dual Dependency-Enhanced Contrastive Learning framework for sparse hypergraph node classification. DDEC aims to recover latent high-order interactions from sparse and noisy observations by jointly leveraging structural and attribute information. Specifically, it introduces an attribute view to complement missing relational information, enhances feature reliability via entropy-guided recalibration, strengthens dual dependencies in a shared relational space through line-graph transformation, and preserves multi-granularity semantic consistency via collaborative contrastive learning at both node and hyperedge levels. Extensive experiments on eight public datasets show that DDEC achieves strong performance on most datasets while exhibiting robust behavior under structural and feature perturbations, although its improvements are limited on some low-connectivity or heterogeneous datasets.

Despite its promising performance, the proposed method still has several limitations that deserve further investigation. First, the current framework relies, to some extent, on the homophily assumption within hyperedges, and its adaptability to strongly heterogeneous hypergraphs remains to be improved. Second, as the scale of the hypergraph increases, dual-space modeling and attention computation may introduce considerable computational overhead. Future work may therefore explore more efficient strategies, such as subgraph sampling, sparse attention, or approximate propagation, to improve scalability. Finally, the present study focuses on static hypergraphs. Extending dual dependency enhancement and collaborative contrastive learning to dynamic hypergraph settings, in order to capture the temporal evolution of high-order relations, is also a promising direction for future research.

## Figures and Tables

**Table 1:** Statistics of the Datasets.

Dataset	*n*	*d*	$m^{s}$	$m^{f}$	$\rho_{e}$	$\rho_{v}$	ζ	*K*	*h*
Cora-C	2708	1433	1579	2708	1.77	3.03	1.25	7	0.8625
Citeseer	3312	3703	1079	3312	1.04	3.20	0.85	6	0.8291
Cora-A	2708	1433	1072	2708	1.69	4.28	0.75	7	0.8763
Zoo	101	16	43	101	17	39.93	5.58	7	0.6558
Mushroom	8124	22	298	8124	5	136.31	0.09	2	0.9554
Actor	16,255	50	10,164	16,255	3.39	5.43	0.31	3	0.6755
Amazon	22,299	111	2090	22,299	0.29	3.10	1.60	5	0.6441
Pokec	14,998	65	2406	14,998	0.37	2.29	0.66	2	0.7225

Note: *n* denotes the number of nodes, *d* denotes the dimensionality of node features, $m^{s}$ and $m^{f}$ denote the numbers of hyperedges in the structural-view and attribute-view hypergraphs, respectively, $\rho_{e}$ denotes the average hyperedge cardinality, $\rho_{v}$ denotes the average node participation, ζ denotes the average number of neighboring hyperedges in the dual space, *K* denotes the number of classes, and *h* denotes the intra-hyperedge label homophily ratio, which measures the proportion of node pairs within the same hyperedge that share the same class label.

**Table 2:** Comparison of classification accuracy (%) between DDEC and baseline methods.

Method	Cora-C	Citeseer	Cora-A	Zoo	Mushroom	Amazon	Pokec	Actor
HGNN	62.99	56.68	70.74	77.78	99.01	25.50	57.34	73.57
HNHN	76.21	67.28	74.88	74.04	97.00	26.34	56.33	71.76
AllSet	76.21	67.83	76.94	65.43	98.48	25.63	56.04	77.12
DHGNN	70.88	63.92	75.91	79.01	OOM	24.31	54.73	63.47
HSL	75.45	65.43	74.17	76.54	99.69	26.12	50.88	**82.97**
TDHNN	68.76	66.64	71.94	41.98	97.09	27.02	57.02	62.46
HyperGCL	73.52	66.82	76.57	66.89	99.46	26.09	53.80	75.44
CHGNN	77.00	68.96	79.95	77.38	98.89	26.44	50.78	66.09
HyGCL-AdT	76.64	70.45	80.25	76.54	**99.77**	25.87	**58.68**	81.37
DPHGNN	49.05	40.20	73.37	80.00	94.83	19.24	55.53	64.27
DDEC	**78.31**	**72.87**	**81.17**	**81.48**	99.54	**27.40**	57.64	80.97
Improve	1.31	2.42	0.92	1.48	−0.23	0.38	−1.04	−2.00

Note: The best results are shown in bold, and the second-best results are underlined. “OOM” indicates that the method runs out of memory. “Improve” denotes the absolute performance gain of DDEC over the strongest baseline on each dataset.

**Table 3:** Variants used in the ablation study of DDEC.

Variant	Feature Recalibration	Line Graph	Attention	Node-Level Loss	Hyperedge-Level Loss	Attribute View
w/o Feature Recalibration	×	✓	✓	✓	✓	✓
w/o Line Graph	✓	×	✓	✓	✓	✓
w/o Attention	✓	✓	×	✓	✓	✓
w/o Node-level Loss	✓	✓	✓	×	✓	✓
w/o Hyperedge-level Loss	✓	✓	✓	✓	×	✓
w/o Attribute View	✓	✓	✓	✓	✓	×
DDEC	✓	✓	✓	✓	✓	✓

Note: ✓ indicates that the corresponding module is used, while × indicates that it is removed.

**Table 4:** Training time (in seconds) of DDEC and representative baselines on five datasets.

Method	Cora-C	Citeseer	Cora-A	Zoo	Mushroom
HyGCL-AdT	60	72	86	31	282.83
DPHGNN	182	220	168	129	4620
DDEC	206	227	123	12	5580

## Data Availability

The data used in this study are publicly available online or are available from the corresponding authors.
